# 

**Published:** 1889-11-16

**Authors:** 


					104 THE HOSPITAL NoYrEMBEK 16, 1889.
NOTICE TO CORRESPONDENTS.
All MS., Letters, Books for Review, and other matters intended for the
Editor, should be addressed The Editor, The Lodge, Porchester
Square, London, W.
Notice to Advertisers and Subscribers.
All Advertisements, Orders for Copies of The Hospital, and Business
Communications should be addressed to The Publisher, not to the Editor,
The Hospital, 139-140, Salisbury-court, Fleet-street, E.C.
Bound Volumes of "The Hospital."
Vol. VI., containing full page portraits of T.R.H. the Prince and
Princess of Wales, and Miss Florence Nightingale, is now ready, 4s., post
free. Cases for binding, may be had of the Publisher, at Is. 6d. each.
To Residents, Secretaries, and Matrons
The Editor will be glad to have the Regulations and each Report as
issued by Asylums, Hospitals, Convalescent and Nursing Institutions, as
well as those of other Charities, and an early copy in duplicate of all
Appeals and Festival Notices, etc., addressed to The Editor, The Lodge
Porchester-xquare, London, W. Any superintendent, secretary, matron,
or other chief official who regularly sends such information may be
registered, on application to the Publisher, to receive free of cost a cover
or binding The Hospital in half-yearly volumes.
Scraps and Gleanings.
Hospital Sunday collections at Tiverton reached ?45.
The Chaplain of Guy's is trying to start a Workhouse Aid Society.
Cholera his disappeared from Ganjam, and relief circles have been
stopped.
Brackley Cottage Hospital treated 38 patients last year. Finances
are satisfactory.
Mrs. Richardson is delivering free lectures on physiology to the
women of Glastonbury.
Viscountess Combermere has left ?28,000 to build a Gibbings Wing
to North Cork Infirmary.
A ball will be held at Brighton on the 21st, in aid of St. John's Con-
valescent Home for Children.
A sum of 40,000f. has been transmitted to M. Pasteur by the Mansion
House Pasteur Institute Fund.
A demonstration of working men in aid of Montagu Cottage Hospital
resulted in a collection of ?15.
The committee of the McAll Mission in Paris will report a deficit of
some ?2,200 in December next.
The annual concert in aid of Warrington Hospital was held on Novem-
ber 1st. About ?50 was taken.
Dr. Grimshaw and Dr. MacCabe have been examined by the Royal
Commission upon Vaccination.
Winter entertainments have begun at Brompton Hospital. Mdlle.
Alice Roselli organised the first programme.
Santa Claus Society gifts should be sent to the secretary, Miss J. K-
Charles, Hillside, Southwood-lane, Highgate.
There were 77 deaths in Tunbridge Wells during September quarter,
being equal to a death-rate of 11'04 per 1,000 per annum.
The German Hospital, Dalston, admitted 13S in-patients during
October ; 25 were accident cases, 21 of them being English.
The Queen has contributed ?25 towards the Royal Victoria Pension
Fund of the Newsvendors' Benevolent and Provident Institution.
Sheffield has raised ?950 for the London Missionary Society in one
year, and Leeds has raised ?650 for the Baptist Missionary Society.
There are now more than 200 Indian ladies studying medicine in the
schools of Bombay, Calcutta, Lahore, Madras, Hyderabad, and Agra.
Mrs. Evans, of Llanelly, has been delivered of four children?three
girls and one boy. The boy and one girl died ; the mother is doing well.
" Her Own Witness" is a play by Dr. G. H. R. Dabbs, which was pro-
duced at the Criterion last week. The plot turns on a case of somnam-
bulism.
The seventh series of Prince's Cinderella dances will be held at
Prince's Hall, Piccadilly, this winter, in aid of the Chelsea Hospital for
Women.
Mr. George Palmer, formerly M.P. for Reading, and head of the
great biscuit firm, has presented a second recreation ground to the
borough.
Cheltenham collected ?395 on Hospital Sunday, against ?475 col-
lected last year. Two or three places of worship have, however, not yet
made their collection.
The library at the new Morley Memorial College, Royal Victoria
Hall, Waterloo-road, has had a valuable gift of a thousand volumes
from Mr. J. Passmore Edwards.
In connection with the Thames Church Mission, a special appeal has
just been issued, signed by the Bishop of London, the late Lord Mayor,
and others, asking for contributions.
At a meeting at Glasgow a subscription was started for the sufferers
by the recent accident at Templeton's Carpet Factory. Subscriptions to
the amount of ?2,385 were announced.
The Hon. Mrs Powys-Keck, who founded and has largely supported
Torquay Western Hospital, has been obliged by ill-health to retire from
the active management of the institution.
A meeting of the Charitable and Voluntary Association, members of
the School Board of London, have decided to provide meals for
necessitous children attending the schools.
There is an epidemic of measles in Cupar, Fife, which is rapidly
spreading. The number of children detained from one of the schools,
which has an average attendance of 330, is now 210.
Great Northern Central Hospital, N. The number of patients
relieved during the month of October was: Medical, 2,099 ; surgical,
2,262 ; total, 4,361; of which 1,371 were new cases, and 325 accidents.
THE half-yearly general meeting of the British Home for Incurables
took place on Thursday, the 14th inst., at Cannon-street Hotel, at 1*
o'clock, the Earl Amherst presiding. Thirteen candidates were elected-
The Bradshawe Lecture will be delivered by Thomas Bryant, Esq., ?n
Thursday, December 5tli, at 5 p.m., the subject of the lecture being
" Colotomy, Lumbar and Iliac, with special reference to the choice of
Operation."
Fleet Surgeon T. G. Wilson, Deputy Inspector-General of Hospitals
and Fleets, retired, was found lying dead in Southsea, lately. He tooK
part in the Egyptian campaign, and wore the Egyptian medal and the
Khedive's bronze star.
Miss Elizabeth Simpson Stone, of Norwich, has presented a
lifeboat to the Gorleston volunteer lifeboat crew, to take the place oi
the lifeboat "Refuge," which was capsized at the entrance of the har-
bour on the 18th of November last.
Mr. Alderman Marsden, the Mayor of Barnsley, on Saturday,
2nd inst., formally presented to the governors of the Beckett Hosprt?
an ambulance carriage, with complete fittings, provided by public sub-
scription, at the cost of upwards of ?130.
In a certain hotel at Aston, near Birmingham, button-holes are Pr?"
vided by the landlord, but close to the tiny nosegays stands a box
which a penny is supposed to be dropt by those who help themselves
flowers. About 10 guineas a year is thus collected and sent to liinniDa'
ham hospitals.

				

